# Vicarious post-traumatic growth in Chinese oncology nurses: A cross-sectional study

**DOI:** 10.1371/journal.pone.0326185

**Published:** 2025-06-18

**Authors:** Jinghan Xu, Dandan Chen, Blandine Lucrece Jongo Fouelefack, Yunxian Zhou

**Affiliations:** School of Nursing, Zhejiang Chinese Medical University, Hangzhou, Zhejiang, China; Ministry of Health, General Health Directorate of Raparin and University of Raparin, IRAQ

## Abstract

In the traditional Chinese culture, vicarious trauma poses a significant threat to oncology nurses who are frequently exposed to death and illness. This exposure can undermine both the physical and mental health of these nurses, potentially affecting team retention. Vicarious post-traumatic growth, a positive outcome of trauma exposure, has been shown to mitigate the adverse effects of vicarious trauma. As a result, fostering vicarious post-traumatic growth is an important area of focus. However, the prevalence of vicarious post-traumatic growth and its influencing factors is limited. The relationship between vicarious post-traumatic growth and vicarious trauma also remains unclear. This study used a cross-sectional survey design. A total of 445 questionnaire were collected between October and December 2023, with 401 valid responses retained for analysis. Participants completed questionnaires that included demographic and work-related variables questionnaire, the Vicarious Trauma Questionnaire (VTQ) and the Chinese-Post Traumatic Growth Inventory (C-PTGI). Descriptive statistics, Pearson correlation analysis, t-tests or ANOVA (F-tests) and multiple regression analysis were conducted to investigate the level of vicarious post-traumatic growth and the modifiable factors among oncology nurses. Multicollinearity diagnostics confirmed no significant collinearity among variables. This study found that Chinese oncology nurses developed high levels of vicarious post-traumatic growth (n = 401,68.67 ± 18.88) based on medium levels of vicarious trauma (n = 401,66.32 ± 22.50). In the multiple regression analysis, vicarious trauma (B = −0.129, 95%CI −0.211 ~ −0.046), social support (B = 7.963, 95%CI 4.680 ~ 11.247), and job satisfaction (B = 7.418, 95%CI 5.444 ~ 9.391) were independently associated with vicarious post-traumatic growth. These findings have important implications for the future implementation of effective interventions to improve the level of vicarious post-traumatic growth. Recommendations include death education, emotional labor strategies training, mindfulness therapy, psychological counseling, and Balint groups, which can improve the level of vicarious post-traumatic growth in Chinese oncology nurses.

## 1 Background

Nurses play a crucial role in healthcare systems worldwide, comprising approximately half of all healthcare professionals. However, prolonged exposure to patient death, suffering, and trauma makes nurses vulnerable to significant indirect distress, a phenomenon known as vicarious trauma (VT) which can hinder their professional performance [1]. The effects of VT are wide-ranging, from psychological disturbances to physical symptoms [2]. If left unaddressed, VT can lead to decreased job satisfaction and diminished professional efficacy [3]. Recent studies indicate that working with trauma can also foster positive transformations, known as vicarious post-traumatic growth (VPTG) [4]. VPTG is the trauma workers’ positive transformation from compassionate involvement with trauma survivors [5]. It manifests as beneficial changes in self-awareness, interpersonal relationships, and life perspectives [6]. VPTG can mitigate the adverse effects of trauma exposure by enhancing emotional resilience, deepening the understanding of personal strength, and improving coping mechanisms, thereby lessening the psychological burden of indirect trauma and enhancing mental health [7]. Research suggests that VPTG can enhance retention rates and improve the quality of their services provided by trauma workers [8].

There is increasing interest in understanding the psychological consequences for individuals exposed to traumatic environments. Much of the literature suggests that VPTG is very similar to post-traumatic growth (PTG) [9], and that indirect exposure to trauma can also lead to personal growth, although this is often overlooked [10]. To date, several studies have explored VPTG in professionals, reporting important work-related benefits or rewards from vicarious trauma [11]. For nurses, work-related VT is inevitable [12]; therefore, stimulating potential positive consequences is essential, encouraging nurses to approach their work with greater resilience and professionalism.

In oncology settings, nurses frequently experience greater stress compared to those in general medical wards. They care for patients requiring long-term treatment, managing complex complications, and often facing substantial patient financial strain [13]. Due to the prolonged and incurable nature of cancer treatment, oncology nurses often develop profound and enduring relationships with patients and frequently confront the reality of death. At the same time, in China, a culture of death avoidance and denial exists [14], where death and illness are often perceived as signs of misfortune or bad luck. This cultural view creates significant challenges for healthcare professionals in addressing death-related issues [15]. Within this traditional Chinese cultural context, such exposures may render Chinese oncology nurses particularly susceptible to the negative impacts of VT, potentially harming their physical and mental health. Currently, the number of cancer patients is growing rapidly in China, where cancer has become the leading cause of death [16]. However, there is a shortage of oncology nurses in the country [17]. Therefore, increasing the incidence of VPTG, and retaining experienced oncology nurses are critical priorities. Prior research has established that social support [18,19] and job satisfaction [20] are significant predictors of VPTG. Crucially, both are recognized as key modifiable variables, suggesting that a deeper understanding of their influence could yield critical insights for developing institutional health policies. However, while these factors have been examined in populations such as emergency nurses and general medical personnel, there is a paucity of evidence regarding their specific effects on oncology nurses, particularly within the Chinese context. Therefore, this study aims to investigate the current levels of VPTG among Chinese oncology nurses and examine whether these modifiable factors constitute promotive determinants of VPTG in Chinese oncology nursing contexts.

## 2 Methods

### 2.1 Study design

To examine the current status of VPTG and its modifiable factors, a cross-sectional survey was conducted from October 2023 to December 2023, in Zhejiang province, China. This cross-sectional study strictly followed the STROBE (Strengthening the Reporting of Observational Studies in Epidemiology) guidelines for observational research [21].

### 2.2 Participants

Participants were selected based on the following inclusion and exclusion criteria. Inclusion criteria required participants to have at least one year of experience in oncology nursing. Exclusion criteria included standardized training nurses, continuing education nurses, or rotating nurses. Additionally, to ensure that VPTG was a result of professional exposure rather than personal trauma, individuals currently experiencing or who had not fully recovered from specific situations were excluded: personal or family member suffering from a significant illness (physical or psychological) or significant accident (such as a car accident or fire), strained family relationships (such as separation or divorce), the death of a family member, involvement in a serious medical error, or exposure to severe workplace violence (including physical assaults or psychological abuse). These events included in our exclusion criteria were based on the Trauma History Screen scale and were informed by prior literature as well as the occupational characteristics of nurses [22–24].

### 2.3 Study sample

The study employed a convenience sampling method. Participants meeting the predefined inclusion and exclusion criteria were recruited via an online platform. To enhance sample diversity and mitigate potential selection bias, recruitment efforts spanned multiple cities within Zhejiang Province. The sample size was determined using G*Power software (version 3.1). Based on a medium effect size of 0.15, α of 0.05, a power of 0.95, and 10 independent variables in the multiple regression model, the minimum required sample size was determined to be 172 nurses. With a 20% inefficiency adjustment, the final required sample size was 207.

### 2.4 Study tools

#### 2.4.1 Demographic and work-related variables questionnaire.

A self-designed questionnaire was used to collect demographic and work-related information, including age, gender, marital status, fertility status, years of experience, job title, level of education, social support, and job satisfaction.

#### 2.4.2 Vicarious Traumatic Questionnaire (VTQ).

This questionnaire, developed by Qingqing Zhang of Zhejiang University, was specifically designed for healthcare workers [25]. It measures the psychological effects of indirect exposure to traumatic events through patient care, focusing on three dimensions of VT: negative affect, avoidance and somatization, negative cognition and alert reaction. The questionnaire consists of 29 items rated on a 5-point Likert scale ranging from 1 = “never” to 5 = “very often”. The levels of VT based on the total score of the VTQ can be categorized as low (29–60), moderate (61–90), high (91–120), and severe (121–145). The VTQ demonstrated acceptable reliability and validity in the present study. Internal consistency was high, with an overall Cronbach’s α of 0.959 and dimensional coefficients of 0.898, 0.958, and 0.923, respectively. Furthermore, confirmatory factor analysis (CFA) supported the instrument’s robust construct validity (see S1 Appendix for detailed psychometric analysis).

#### 2.4.3 The Chinese-post traumatic growth inventory (C-PTGI).

In the present study, the C-PTGI, originally developed by Tedeschi and Calhoun and subsequently translated by Ji Wang, was utilized to assess VPTG levels among oncology nurses in Zhejiang Province. The C-PTGI is a 20-item instrument employing a 6-point Likert scale (0 = “none” to 5 = “very large”) for item responses. In the current study, the C-PTGI demonstrated excellent internal consistency, with an overall Cronbach’s α coefficient of 0.965 (refer to S1 Appendix for a detailed psychometric analysis). The selection of the C-PTGI was methodologically justified on several grounds. Firstly, given that VPTG and PTG share the core mechanism of cognitive restructuring in fostering positive post-traumatic adaptation, the original PTGI has been extensively utilized to measure VPTG [8,26,27]. Secondly, in the absence of a culturally adapted and psychometrically validated instrument specifically designed for assessing VPTG within Chinese populations, the C-PTGI represented a sound alternative. This approach is further supported by previous validation studies of the PTGI, which have indicated superior model fit for a single-factor structure compared to the original five-factor model, thereby recommending the use of total scores rather than subscale scores [28–31]. Therefore, consistent with this empirical evidence and the specific objectives of the current investigation, the total C-PTGI score was employed as the primary metric for this instrument. The total score of the C-PTGI allows for categorization of VPTG levels as low (0–59), moderate (60–65), and high (66–100) [32].

### 2.5 Data collection

This study focused on oncology nurses in Zhejiang Province as participants. Data collection was conducted through an online survey created using the WenJuanXing platform and distributed via WeChat, a popular social media application. To ensure data integrity, each IP address was restricted to a single submission. Participants were required to complete a questionnaire that included demographic and work-related variables, as well as the VTQ and the C-PTGI. To maintain the quality of responses, comprehensive instructions were provided at the beginning of the questionnaire. These instructions elucidated the study’s purpose, guided participants on how to answer questions, and assured them of data anonymity. Anonymity also can minimize social desirability bias, which may lead to over-reporting of VPTG by respondents and thus compromise the validity of the results. Participants were informed that their responses would be confidential and would not be linked to their personal identities. A total of 445 questionnaire responses were initially collected. To ensure data quality, those completed in less than 116 seconds (less than 2 seconds per item across 58 items) or with the same answers for all items were excluded [33,34]. This resulted in 401 valid questionnaires, yielding a valid response rate of 89.1%.

### 2.6 Data analysis

Data analysis was conducted using SPSS Statistics 26.0. The dataset used for analysis is available in Supporting Information as S1 Data. Descriptive statistics were employed, with means and standard deviations used for measurement data, and frequencies and percentages for categorical data. Pearson correlation analysis was utilized to examine the relationship between VT and VPTG. To determine statistically significant differences between various factors and VPTG, t-tests or ANOVA (F-tests) were used in univariate analysis. Subsequently, multiple regression analysis was performed to identify factors influencing VPTG, with evaluation of multicollinearity using variance inflation factors (VIFs). *P*-values were two-sides, and *P* < 0.05 indicated statistical significance.

### 2.7 Ethical consideration

This study received ethical approval from Zhejiang Chinese Medical University’s ethical committee, where the researchers are based (20230922−6).

## 3 Results

### 3.1 Participant characteristics

The demographic and work-related characteristics of the participants are presented in Table 1. The majority of the participants were aged 30–39 years (41.65%) and female (98.5%). The majority of participants held intermediate positions (47.63%), were bachelor degree (90.28%). The remaining details are shown in Table 1. Furthermore, Table 1 also presents the levels of VT and VPTG among participants. The mean C-PTGI score for oncology nurses was 68.67 (SD = 18.88), while the mean VTQ score among oncology nurses was 66.32 (SD = 22.50).

**Table 1:** Characteristics of Participants (n = 401).

Characteristics		N (%)/ mean±standard deviation
Age (years)	20-29	123 (30.67)
	30-39	167 (41.65)
	≥40	111 (27.68)
Gender	Man	6 (1.50)
	Woman	395 (98.50)
Years of experience	1-5	99 (24.69)
	6-15	186 (46.39)
	>15	116 (28.92)
Job title	Junior	178 (44.38)
	Intermediate	191 (47.63)
	Associate chief or Chief	32 (7.99)
Level of education	Associate degree	28 (6.98)
	Bachelor degree	362 (90.28)
	Master degree	11 (2.74)
Marital status	Unmarried	125 (31.17)
	Married	276 (68.83)
Fertility status	Not given birth	147 (36.65)
	Given birth	254 (63.35)
Social support	Low	14 (3.50)
	Medium	198 (49.37)
	High	189 (47.13)
Job satisfaction	Dissatisfied	40 (9.99)
	Commonly	157 (39.15)
	Satisfied	204 (50.86)
VTQ C-PTGI		66.32 ± 22.50 68.67 ± 18.88

### 3.2 Relationship between VT and VPTG

Pearson correlation analyses were conducted to examine the presence of a significant linear relationship between VTQ with its dimensions and C-PTGI, as shown in Table 2. A small but significant negative correlation was found between overall VTQ scores and C-PTGI scores (r = −0.127, *p* < 0.05). Specific dimensions of VT such as “avoidance and somatization” (r = −0.189, *p* < 0.01) and “negative cognition and alert reaction” (r = −0.116, *p* < 0.05) also demonstrated negative associations with VPTG.

**Table 2:** Correlation between C-PTGI and VTQ (N = 401).

	C-PTGI
VTQ	−0.127^*^
Negative affect	0.036
Avoidance and somatization	−0.189**
Negative cognition and alert reaction	−0.116*

Note: * *p* < 0.05; ** *p* < 0.01.

### 3.3 Factors influencing of VPTG

Table 3 presents the means, standard deviations, and statistical comparisons (t/F and p-values) of VPTG scores across various demographic and work-related factors among oncology nurses. The analysis revealed several significant findings: marital status (t = −2.222, *p* = 0.027) and fertility status (t = −2.766, *p* = 0.006) were significant predictors of VPTG scores. Furthermore, significant differences in VPTG scores were observed based on levels of social support (F = 15.053, *p* < 0.001) and job satisfaction (F = 26.169, *p* < 0.001). Nurses reporting high social support or high job satisfaction demonstrated higher VPTG scores.

**Table 3:** Summary of Demographic and Work-Related of VPTG Scores (N = 401).

Characteristics		Mean (SD)	t/F	*p*
Age (years)	20-29	66.16(20.99)	1.738	0.177
	30-39	70.31(18.23)		
	≥40	68.98 (17.16)		
Years of experience	1-5	66.13(20.53)	1.192	0.305
	6-15	69.46 (19.54)		
	>15	69.58(16.08)		
Job title	Junior	68.24(18.91)	2.757	0.065
	Intermediate	67.83(19.54)		
	Associate chief or Chief	76.13 (12.38)		
Level of education	Associate	67.11(19.97)	0.170	0.844
	Bachelor	68.85(18.73)		
	Master	66.73(22.46)		
Marital status	Unmarried	65.36(21.10)	−2.222	0.027
	Married	70.17(17.62)		
Fertility status	Not given birth	65.10(20.89)	−2.766	0.006
	Given birth	70.74(12.32)		
Social support	Low	52.21(27.72)	15.053	<0.001
	Medium	65.39(17.14)^a^		
	High	73.33(18.54)^ab^		
Job satisfaction	Dissatisfied	57.87 (23.90)	26.169	<0.001
	Commonly	63.46(17.01)^c^		
	Satisfied	74.79 (16.98)^de^		

Note: a compared with low social support group: *p* < 0.01; b compared with medium social support group: *p* < 0.01; c compared with dissatisfied group: *p* > 0.05; d compared with dissatisfied group: *p* < 0.01; e compared with commonly group: *p* < 0.01.

Prior to interpreting multiple regression results, collinearity diagnostics were conducted to verify the stability of coefficient estimates. Collinearity diagnostics using variance inflation factors (VIF) indicated no concerning multicollinearity among predictors, with all VIF values falling below the threshold of 10 [35] (S1 Table). This suggests that regression coefficient estimates remain stable and interpretable without requiring variable exclusion or transformation. The multiple regression analysis, summarized in Table 4, identified intervention-targetable factors for VPTG, including social support, job satisfaction and VT. VT experiences were negatively associated with VPTG scores (B = −0.129, 95%CI −0.211 ~ −0.046) in the model 1. Nurses who have higher levels of social support were positively associated with higher VPTG scores (B = 7.963, 95%CI 4.680 ~ 11.247) in the model 2. Greater job satisfaction was also significantly associated with higher VPTG scores (B = 7.418, 95%CI 5.444 ~ 9.391) in the model 3.

**Table 4:** Multiple Regression Analysis of Factors Associated with VPTG.

Variables	Model 1		Model 2		Model 3	
	B(95%CI)	*P*	B(95%CI)	*P*	B(95%CI)	*P*
Constant	74.402(65.599, 83.206)	<0.001	48.046(37.985, 58.106)	<0.001	39.794(30.213, 49.375)	<0.001
Age: 30–39 y	3.013(−5.841, 11.866)	0.504	2.195(−6.490, 10.879)	0.620	−1.747(−10.171, 6.676)	0.684
Age: ≥ 40 y	−2.297(−14.644,10.051)	0.715	−3.142(−15.267, 8.983)	0.611	−5.723(−17.419, 5.973)	0.337
Job Experience: 6-15y	−1.643(−10.585,7.300)	0.718	−1.211(−10.010, 7.587)	0.787	1.130(−7.377, 9.637)	0.794
Job Experience: ≥ 15y	−0.292(−12.625,12.042)	0.963	−0.064(−12.196, 12.067)	0.992	1.119(−10.573, 12.810)	0.851
Job title: Intermediate	−4.637(−10.243,0.969)	0.105	−3.040(−8.505, 2.425)	0.275	−2.397(−7.667, 2.873)	0.372
Job title: Associate chief or chief nurse	5.288(−4.196, 14.773)	0.274	4.728(−4.604, 14.060)	0.320	5.449(−3.524, 14.422)	0.233
Education: Bachelor degree	−0.306(−7.994, 7.383)	0.938	−0.388(−7.950, 7.174)	0.920	1.148(−6.141, 8.437)	0.757
Education: Master degree	−1.392(−14.829,12.044)	0.839	−4.038(−17.268, 9.192)	0.549	−2.425(−15.150, 10.300)	0.708
Marital status	0.252(−7.961, 8.466)	0.952	−1.325(−9.420, 6.769)	0.748	−1.693(−9.487, 6.101)	0.670
Fertility status	7.810(−0.447, 16.066)	0.064	6.635(−1.496, 14.766)	0.109	8.207(0.381, 16.032)	0.040
VT	−0.129(−0.211, −0.046)	0.002				
Social support			7.963(4.680, 11.247)	<0.001		
Job satisfaction					7.418(5.444, 9.391)	<0.001

Note: Model 1,2,3: adjusted for age, job experience, job title, education, marital status and fertility status.

## 4 Discussion

This study investigated the current status and influencing factors of VPTG among oncology nurses in Zhejiang Province. Additionally, it elucidated the relationship between VT and VPTG. The findings aim to provide guidance for nursing managers to enhance the professional development of oncology nurses, maximize the positive effects of VPTG, improve nurses’ well-being, and reduce turnover rates.

According to the classification criteria, the oncology nurses in this study exhibited high levels of VPTG, with a mean C-PTGI score of 68.67 (SD = 18.88). This aligns closely from Yaakubov’s findings [36], who reported an average PTG score of 54.09 (SD = 22.07) for emergency department nurses. Another study also reported a high level of VPTG (M = 70.53, SD = 17.26) in front-line nurses during COVID-19 [37]. These results suggest that oncology nurses can experience high levels of VPTG as a response to VT. However, due to the complexity of factors and internal mechanisms influencing VPTG occurrence, health professionals in different settings may experience varying levels of VPTG. Lower levels of VPTG are less frequently reported, possibly due to publication bias.

Levels of trauma exposure and VT also influence VPTG. Previous studies have shown that community-based outpatient nurses report moderate levels of VPTG [38], while social workers serving in non-domestic violence settings exhibit lower VPTG levels compared to those working in domestic violence fields [39]. In this study, oncology nurses developed high levels of VPTG based on medium levels of VT, and the two showed a negative correlation (B = −0.129, 95%CI −0.211 ~ −0.046) in the regression model. This finding is consistent with previous research that suggests moderate vicarious trauma exposure is strongly associated with high levels of VPTG [40]. The relationship between VT and VPTG remains uncertain. Some studies propose linear relationship, while others suggest a curvilinear inverted “U” shaped curve. According to Joseph’s post-traumatic stress threshold effect, trauma and growth can coexist, but growth is unlikely to occur at very low or very high trauma levels [41]. For growth to occur, the traumatic event must be sufficiently powerful to challenge existing thoughts patterns, prompting individuals to process and adapt to a new reality. However, the level of trauma should not exceed the healthcare worker’s capacity for adaptation [42]. It appears that VPTG correlates with VT to some extent, but as VPTG stabilizes, a negative correlation between the two may emerge. This study also revealed that among the three dimensions of VTQ, “ avoidance and somatization “ (r = −0.189, *p* < 0.01) and “ negative cognition and alert reaction “ (r = −0.116, *p* < 0.05) dimensions also showed negative correlations with VPTG. A previous survey by original scale author on VT among healthcare workers found that vicarious trauma generally registered at a medium level (M = 78.94, SD = 26.06). It was noted that the average score for a single item in the dimension of “ negative affect “ was the highest [25]. The three dimensions of the scale encompass emotion, physiological response and cognition. It may be that emotional changes and fluctuations are more likely to occur than cognitive and behavioral changes. Therefore, the results of this study indicate that the dimensions of “ avoidance and somatization “ and “ negative cognition and alert reaction “ are correlated with VPTG, which is consistent with the negative correlation between VT and VPTG (r = −0.127, *p* < 0.05). Understanding the relationship between VT and VPTG can help nurses and nursing managers recognize that while a certain level of VT may serve as a catalyst for growth, excessive stress could obstruct this process by impairing coping mechanisms and emotional resilience. It is therefore recommended to implement training programs, such as emotional labor strategies, to enhance nurses’ psychological resilience and reduce the negative impact of VT, ultimately fostering higher VPTG levels [43].

Cultural factors also play a significant role in both VT and VPTG. Chinese oncology nurses face unique social-cultural challenges. In China, there is a death avoidance and death-denying culture [14]. This cultural factor affects how nurses experience patients’ death [44] and can exacerbate the negative impacts of VT [45]. Under the influence of such sociocultural perspectives, nurses often perceive death negatively, which increases their susceptibility to VT and complicates the promotion of VPTG. Chinese oncology nurses should not be viewed merely as passive recipients of their culture. Rather, the social value of caring for oncology patients should be promoted as a virtue, transforming the death-avoidant culture. Therefore, it is recommended that death education be integrated into the training of Chinese oncology nurses [46]. This could help them derive positive meaning from their experiences with VT, thereby fostering the occurrence of VPTG. However, Chinese culture, particularly its emphasis on collectivism and Confucian values, may also facilitate VPTG. Compared with individualism in Western countries, collectivism in Chinese culture may also enable oncology nurses to seek social support more effectively within groups, thereby facilitating the development of VPTG [47]. Historically, many Chinese philosophers have emphasized the importance of resilience and perseverance in their discourses, highlighting the positive value of adversity and the capacity of individuals to transcend challenges [48]. Studies indicate that the concept of perseverance in Confucian culture is associated with indicators of positive mental health, and such perseverance may contribute to an individual’s cultivation of positive well-being and life perspectives [48]. Resilience enables individuals to respond more effectively when coping with trauma [49]. Chinese oncology nurses, potentially influenced by Confucian culture, may demonstrate enhanced performance in VPTG. Future research should further investigate the impact of cultural factors on the development of VPTG.

The multiple regression analysis identified key modifiable factors for VPTG. The results of multiple linear regression analysis in this study demonstrated that social support (B = 7.963, 95%CI 4.680 ~ 11.247), job satisfaction (B = 7.418, 95%CI 5.444 ~ 9.391) and VT level (B = −0.129, 95%CI −0.211 ~ −0.046) were significantly correlated with VPTG. Previous studies have identified social support as both a protective factor against VT and a promoting factor for VPTG [8]. Regarding the relationship between social support and VPTG, some studies using structural equation models show that social support can directly affect VPTG (β = 0.25, *p* = 0.001) [19], while others suggest that the direct path between the two is not significant and is mediated through resilience and cognitive effects [7]. Following trauma exposure, social support can enhance social resources and develop coping skills by fostering interpersonal relationships [50]. Social support includes help from supervisors, coworkers, family, and friends. For healthcare workers, support from coworkers is particularly beneficial due to shared experiences [51], making it essential for oncology nurses to provide mutual support to each other. Support from nursing managers is also a promoter of VPTG, which aligns with findings from another study on VT and VPTG [26]. Some research indicates that Chinese nurses experience VPTG more prominently through professional interactions and workplace support than through changes in worldview or self-perception, underscoring the role of the workplace in enhancing VPTG [43]. However, in Chinese culture, emotional restraint and modesty are highly valued, leading to less initiative in expressing emotions. Research on PTG suggests that suppressing emotional expression negatively predicts PTG, whereas social support facilitates PTG [52]. Therefore, nursing managers should foster communication among nurses through psychological counseling, Balint groups, and other means, guiding Chinese oncology nurses to express negative emotions, explore the positive effects of VT, and promote VPTG. Social support satisfies relational needs and provides access to additional resources, which can help foster VPTG [53]. Nurses can also seek support from other sources, such as family, to enhance VPTG. Furthermore, social support from nursing managers can improve nurses’ job satisfaction [54], thereby enhancing VPTG levels. Consequently, nursing managers should prioritize providing adequate social support to nurses to generate positive effects. This study reveals a significant correlation between job satisfaction and VPTG levels. Job satisfaction is an experienced pleasure of accomplishing work and a positive emotional situation acquired by employees as a result of evaluating their job [55]. While few studies have directly examined the correlation between job satisfaction and VPTG, some research indicates that low job satisfaction among nurses is associated with risks such as burnout and compassion fatigue [55,56]. Additionally, one study indicated that nurses’ job satisfaction is associated with the level of nursing care provided; more missed care corresponds to greater dissatisfaction among nurses [57]. These findings suggest that higher job satisfaction fosters a positive work attitude, which may explain its role in promoting VPTG. Therefore, it is recommended that nursing managers focus on creating a supportive work environment to enhance nurses’ job satisfaction and elevate their VPTG levels. However, job satisfaction is influenced by multiple factors such as voluntary choice of the nursing profession, organizational support, and organizational climate [58]. High level of VPTG is likely an outcome of the synergistic effects of these contributing elements. Therefore, further exploration is needed to investigate the relationship between job satisfaction and VPTG. The organizational climate refers to the subjective perceptions and feelings of members regarding the overall internal environment of their organization and serves as a bridge between the organizational system and the behaviors of individuals within it, significantly influencing their psychological states and actions [59]. Research shows that a harmonious, fair, and supportive organizational climate can alleviate burnout and improve job satisfaction [60]. It is recommended that nursing administrators provide nurses with greater respect, encourage the expression of negative emotions, foster a positive and open organizational climate, assist staff in coping with job demands, and ultimately facilitate the development of VPTG.

In line with the “Healthy China 2030” strategy, establishing institutional or national psychological support programs, including subsidized grief counseling for oncology nurses, could help build a resilient nursing workforce essential for advancing China’s cancer care. Future researchers are recommended to conduct longitudinal studies and qualitative research. Longitudinal research tracking nurses across different career stages is essential to capture the dynamic changes in VPTG and to identify the factors that influence its development, which would further enhance our knowledge in this area. Additionally, qualitative studies could be conducted to gain deeper insights into nurses’ experiences of VPTG, providing a more comprehensive understanding of this complex phenomenon.

## 5 Limitation

There are several limitations in this study. First, this study employs a cross-sectional survey design. Due to the inherent limitations of this methodology, the causal relationships between VPTG and influencing factors cannot be determined. Moreover, the data collection is confined to a specific time period, thus failing to reflect long-term dynamic changes. It is advisable to adopt a longitudinal investigation design in future research to address this issue. Second, the data were collected from a single province within China. Acknowledging the diverse economic and cultural backgrounds across different regions, the study’s results may not be fully generalizable to oncology nurses in other areas. It is recommended that future research should expand the scope of investigation to various settings to enhance generalizability. Third, reliance on self-reported data introduces the potential for response bias. At the same time, the use of the C-PTGI, rather than a VPTG-specific instrument, may not fully capture vicarious growth characteristics. Additionally, the exclusion of nurses with recent significant personal trauma, while necessary to isolate VPTG from personal PTG, may have introduced selection bias, potentially underrepresenting the full spectrum of growth experiences or influencing the observed VPTG levels within the sampled population. Finally, due to resource limitations, this study utilized a convenient online platform for collecting questionnaire data, which might also lead to information bias. To ensure high-quality data, future research should consider employing offline data collection methods.

## 6 Conclusion

This study aimed to investigate the level of VPTG among oncology nurses in China and its influencing factors. The results of this study exhibited that Chinese oncology nurses had a high level of VPTG, with social support, job satisfaction, and VT significantly correlated with VPTG. This study provides new insights into how oncology nurses achieve growth in the face of work-related VT, highlighting the important role of relevant influencing factors in the occurrence of VPTG. These findings have important implications for the future implementation of effective interventions to support the physical and mental health of oncology nurses. Nursing managers are encouraged to implement evidence-based strategies including death education, emotional labor strategies training, mindfulness therapy, psychological counseling, and Balint groups. In alignment with national strategies, institutional-level psychological rehabilitation subsidies should prioritize grief counseling access to enhance the VPTG level of Chinese oncology nurses.

## Supporting information

S1 Table Collinear diagnosis of included variables. (DOCX)

S1 Appendix The psychometric analysis of the VTQ and C-PTGI. (DOCX)

S1 Data Original data. (XLSX)

## References

[1] Mairean C, Cimpoesu D, Turliuc MN. The effects of traumatic situations on emergency medicine practitioners. Revista De Cercetare St Interventie Sociala. 2014;44:279–90. doi: 10.13125/44387

[2] Hartley H, Wright DK, Vanderspank-Wright B, Grassau P, Murray MA. Dead on the table: A theoretical expansion of the vicarious trauma that operating room clinicians experience when their patients die. Death Stud. 2019;43(5):301–10. doi: 10.1080/07481187.2018.1461711 29757122

[3] Sui X-C, Padmanabhanunni A. Vicarious trauma: The psychological impact of working with survivors of trauma for South African psychologists. J Psychol Afr. 2016;26(2):127–33. doi: 10.1080/14330237.2016.1163894

[4] Beck CT, Casavant S. Vicarious Posttraumatic Growth in NICU Nurses. Adv Neonatal Care. 2020;20(4):324–32. doi: 10.1097/ANC.0000000000000689 31895140

[5] Arnold D, Calhoun LG, Tedeschi R, Cann A. Vicarious Posttraumatic Growth in Psychotherapy. J Humanist Psychol. 2005;45(2):239–63. doi: 10.1177/0022167805274729

[6] Yu H, Jiang A, Shen J. Prevalence and predictors of compassion fatigue, burnout and compassion satisfaction among oncology nurses: A cross-sectional survey. Int J Nurs Stud. 2016;57:28–38. doi: 10.1016/j.ijnurstu.2016.01.012 27045562

[7] Jiang Y, Qiao T, Zhang Y, Wu Y, Gong Y. Social support and vicarious posttraumatic growth among psychological hotline counselors during COVID-19: the role of resilience and cognitive reappraisal. Health Psychol Behav Med. 2023;11(1):2274550. doi: 10.1080/21642850.2023.2274550 38532890 PMC10964826

[8] Wegrzyn A. Examining predictors of vicarious posttraumatic growth among sexual assault service providers in rape crisis centers. PhD Thesis. DePaul University; 2023. Available from: https://via.library.depaul.edu/csh_etd/464

[9] Hyatt-Burkhart D. The Experience of Vicarious Posttraumatic Growth in Mental Health Workers. J Loss Trauma. 2014;19(5):452–61. doi: 10.1080/15325024.2013.797268

[10] Amowe EO. Trauma coping strategies and posttraumatic growth among catholic religious men and women survivors of kidnapping in southern ecclesiastical provinces of nigeria. MsD Thesis. University of Eastern Africa; 2022.

[11] Tsirimokou A, Kloess JA, Dhinse SK. Vicarious Post-traumatic Growth in Professionals Exposed to Traumatogenic Material: A Systematic Literature Review. Trauma Violence Abuse. 2023;24(3):1848–66. doi: 10.1177/15248380221082079 35487902 PMC10240641

[12] Wu S, Singh-Carlson S, Odell A, Reynolds G, Su Y. Compassion Fatigue, Burnout, and Compassion Satisfaction Among Oncology Nurses in the United States and Canada. Oncol Nurs Forum. 2016;43(4):E161-9. doi: 10.1188/16.ONF.E161-E169 27314199

[13] Liu M, Liu L, Lv Z, Mao Y, Liu Y. Career adaptability among new oncology nurses: A longitudinal exploration. Int Nurs Rev. 2025;72(1):e12988. doi: 10.1111/inr.12988 38778677

[14] Dong F, Zheng R, Chen X, Wang Y, Zhou H, Sun R. Caring for dying cancer patients in the Chinese cultural context: A qualitative study from the perspectives of physicians and nurses. Eur J Oncol Nurs. 2016;21:189–96. doi: 10.1016/j.ejon.2015.10.003 26547883

[15] Tu J, Shen M, Li Z. When cultural values meets professional values: a qualitative study of chinese nurses’ attitudes and experiences concerning death. BMC Palliat Care. 2022;21(1):181. doi: 10.1186/s12904-022-01067-3 36242029 PMC9561326

[16] Xia C, Dong X, Li H, Cao M, Sun D, He S, et al. Cancer statistics in China and United States, 2022: profiles, trends, and determinants. Chin Med J (Engl). 2022;135(5):584–90. doi: 10.1097/CM9.0000000000002108 35143424 PMC8920425

[17] Li Y, Yu W, Li L, Yao Q, Jiang K, Zhu T, et al. Oncology nursing on the move: a contemporary issue on Chinese oncology nursing in cancer care. Front Public Health. 2023;11:1061572. doi: 10.3389/fpubh.2023.1061572 37181711 PMC10173744

[18] Xu X, Hu M-L, Song Y, Lu Z-X, Chen Y-Q, Wu D-X, et al. Effect of Positive Psychological Intervention on Posttraumatic Growth among Primary Healthcare Workers in China: A Preliminary Prospective Study. Sci Rep. 2016;6:39189. doi: 10.1038/srep39189 27995960 PMC5171914

[19] Kang X, Fang Y, Li S, Liu Y, Zhao D, Feng X, et al. The Benefits of Indirect Exposure to Trauma: The Relationships among Vicarious Posttraumatic Growth, Social Support, and Resilience in Ambulance Personnel in China. Psychiatry Investig. 2018;15(5):452–9. doi: 10.30773/pi.2017.11.08.1 29695152 PMC5976003

[20] Ogińska-Bulik N, Gurowiec PJ, Michalska P, Kędra E. Prevalence and determinants of secondary posttraumatic growth following trauma work among medical personnel: a cross sectional study. Eur J Psychotraumatol. 2021;12(1):1876382. doi: 10.1080/20008198.2021.1876382 33968315 PMC8079025

[21] von Elm E, Altman DG, Egger M, Pocock SJ, Gøtzsche PC, Vandenbroucke JP, et al. The Strengthening the Reporting of Observational Studies in Epidemiology (STROBE) statement: guidelines for reporting observational studies. J Clin Epidemiol. 2008;61(4):344–9. doi: 10.1016/j.jclinepi.2007.11.008 18313558

[22] Zhuo L. The research on current status and influencing factors of post-traumatic growth on second victims after medical adverse events. MsD thesis. Zhongnan University; 2023. Available from: https://www.cnki.net

[23] Carlson EB, Smith SR, Palmieri PA, Dalenberg C, Ruzek JI, Kimerling R, et al. Development and validation of a brief self-report measure of trauma exposure: the Trauma History Screen. Psychol Assess. 2011;23(2):463–77. doi: 10.1037/a0022294 21517189 PMC3115408

[24] Zhang Y, Gao Y, Zhang N, Xu K, Zhao S. The relationship between dyadic coping and post-traumatic growth in breast cancer patients and spouses: based on potential profile analysis. BMC Psychiatry. 2024;24(1):860. doi: 10.1186/s12888-024-06289-8 39614210 PMC11606235

[25] Zhang QQ. Development of the scale and the influencing mechanism of medics’ vicarious traumatization. MsD Thesis. Zhejiang University; 2022. Available from: https://www.wanfangdata.com.cn

[26] Cosden M, Sanford A, Koch LM, Lepore CE. Vicarious trauma and vicarious posttraumatic growth among substance abuse treatment providers. Subst Abus. 2016;37(4):619–24. doi: 10.1080/08897077.2016.1181695 27163485

[27] Cleary E, Curran D, Dyer K, Simms J, Hanna D. Contributing factors to secondary traumatic stress and vicarious posttraumatic growth in therapists. J Trauma Stress. 2024;37(1):103–12. doi: 10.1002/jts.22995 37985165

[28] Konkolÿ Thege B, Kovács É, Balog P. A bifactor model of the Posttraumatic Growth Inventory. Health Psychol Behav Med. 2014;2(1):529–40. doi: 10.1080/21642850.2014.905208 25750800 PMC4346070

[29] Osei-Bonsu PE, Weaver TL, Eisen SV, Vander Wal JS. Posttraumatic Growth Inventory: Factor Structure in the Context of DSM-IV Traumatic Events. ISRN Psychiatry. 2011;2012:937582. doi: 10.5402/2012/937582 23738193 PMC3667636

[30] Widows MR, Jacobsen PB, Booth-Jones M, Fields KK. Predictors of posttraumatic growth following bone marrow transplantation for cancer. Health Psychol. 2005;24(3):266–73. doi: 10.1037/0278-6133.24.3.266 15898862

[31] Jozefiaková B, Kaščáková N, Adamkovič M, Hašto J, Tavel P. Posttraumatic Growth and Its Measurement: A Closer Look at the PTGI’s Psychometric Properties and Structure. Front Psychol. 2022;13:801812. doi: 10.3389/fpsyg.2022.801812 36092073 PMC9449875

[32] Wang J. Development of posttraumatic growth inventory and its norm for patient with accidental trauma. MsD Thesis. Second Military Medical University; 2011. Available from: https://www.cnki.net

[33] Meade AW, Craig SB. Identifying careless responses in survey data. Psychol Methods. 2012;17(3):437–55. doi: 10.1037/a0028085 22506584

[34] Huang JL, Curran PG, Keeney J, Poposki EM, DeShon RP. Detecting and Deterring Insufficient Effort Responding to Surveys. J Bus Psychol. 2011;27(1):99–114. doi: 10.1007/s10869-011-9231-8

[35] Dormann CF, Elith J, Bacher S, Buchmann C, Carl G, Carré G, et al. Collinearity: a review of methods to deal with it and a simulation study evaluating their performance. Ecography. 2012;36(1):27–46. doi: 10.1111/j.1600-0587.2012.07348.x

[36] Yaakubov L, Hoffman Y, Rosenbloom T. Secondary traumatic stress, vicarious posttraumatic growth and their association in emergency room physicians and nurses. Eur J Psychotraumatol. 2020;11(1):1830462. doi: 10.1080/20008198.2020.1830462 33408806 PMC7747932

[37] Cui PP, Wang PP, Wang K, Ping Z, Wang P, Chen C. Post-traumatic growth and influencing factors among frontline nurses fighting against COVID-19. Occup Environ Med. 2021;78(2):129–35. doi: 10.1136/oemed-2020-106540 33060188

[38] Zerach G, Shalev TB-I. The relations between violence exposure, posttraumatic stress symptoms, secondary traumatization, vicarious post traumatic growth and illness attribution among psychiatric nurses. Arch Psychiatr Nurs. 2015;29(3):135–42. doi: 10.1016/j.apnu.2015.01.002 26001711

[39] Ben-Porat A, Itzhaky H. Implications of Treating Family Violence for the Therapist: Secondary Traumatization, Vicarious Traumatization, and Growth. J Fam Viol. 2009;24(7):507–15. doi: 10.1007/s10896-009-9249-0

[40] Dar IA, Iqbal N. Beyond linear evidence: The curvilinear relationship between secondary traumatic stress and vicarious posttraumatic growth among healthcare professionals. Stress Health. 2020;36(2):203–12. doi: 10.1002/smi.2932 31994272

[41] Joseph S, Murphy D, Regel S. An affective-cognitive processing model of post-traumatic growth. Clin Psychol Psychother. 2012;19(4):316–25. doi: 10.1002/cpp.1798 22610981

[42] Ogińska-Bulik N, Michalska P. The role of empathy and cognitive trauma processing in the occurrence of professional posttraumatic growth among women working with victims of violence. Int J Occup Med Environ Health. 2022;35(6):679–92. doi: 10.13075/ijomeh.1896.01945 35993797 PMC10464719

[43] Cai Y, Li Y, Zou J, Zhang J, Luo W, Zhang J, et al. Cross-cultural adaptation and reliability of the inventory of vicarious posttraumatic growth and research of its influencing factors: a cross-sectional study. BMC Nurs. 2024;23(1):763. doi: 10.1186/s12912-024-02435-5 39420316 PMC11487754

[44] Shorey S, André B, Lopez V. The experiences and needs of healthcare professionals facing perinatal death: A scoping review. Int J Nurs Stud. 2017;68:25–39. doi: 10.1016/j.ijnurstu.2016.12.007 28063339

[45] Zheng R-S, Guo Q-H, Dong F-Q, Owens RG. Chinese oncology nurses’ experience on caring for dying patients who are on their final days: a qualitative study. Int J Nurs Stud. 2015;52(1):288–96. doi: 10.1016/j.ijnurstu.2014.09.009 25445033

[46] Zhu Y, Bai Y, Wang A, Liu Y, Gao Q, Zeng Z. Effects of a death education based on narrative pedagogy in a palliative care course among Chinese nursing students. Front Public Health. 2023;11:1194460. doi: 10.3389/fpubh.2023.1194460 38026299 PMC10665499

[47] Tsai W, Lau AS. Cultural differences in emotion regulation during self-reflection on negative personal experiences. Cogn Emot. 2013;27(3):416–29. doi: 10.1080/02699931.2012.715080 22916683

[48] Mak WWS, Ng ISW, Wong CCY, Law RW. Resilience Style Questionnaire: Development and Validation Among College Students and Cardiac Patients in Hong Kong. Assessment. 2016;26(4):706–25. doi: 10.1177/1073191116683798 28006974

[49] Masten AS, Narayan AJ. Child development in the context of disaster, war, and terrorism: pathways of risk and resilience. Annu Rev Psychol. 2012;63:227–57. doi: 10.1146/annurev-psych-120710-100356 21943168 PMC5858878

[50] Manning-Jones S, de Terte I. Secondary traumatic stress, vicarious posttraumatic growth, and coping among health professionals: A comparison study. NZ J Psychol. 2016;45(1):20–9.

[51] Geyer LT, Cunningham T, Rastorguieva K, Ritenour CWM. An Abundance Mindset Approach to Support Nurse Well-Being: The Feasibility of Peer Support. Nurse Lead. 2023:10.1016/j.mnl.2023.03.013. doi: 10.1016/j.mnl.2023.03.013 37361414 PMC10163375

[52] Zhou X, Wu X, Zhen R. Understanding the relationship between social support and posttraumatic stress disorder/posttraumatic growth among adolescents after Ya’an earthquake: The role of emotion regulation. Psychol Trauma. 2017;9(2):214–21. doi: 10.1037/tra0000213 27736141

[53] Marroquín B, Nolen-Hoeksema S. Emotion regulation and depressive symptoms: Close relationships as social context and influence. J Pers Soc Psychol. 2015;109(5):836–55. doi: 10.1037/pspi0000034 26479366 PMC4616056

[54] Orgambídez A, Almeida H. Social support, role clarity and job satisfaction: a successful combination for nurses. Int Nurs Rev. 2020;67(3):380–6. doi: 10.1111/inr.12591 32436283

[55] Akman O, Ozturk C, Bektas M, Ayar D, Armstrong MA. Job satisfaction and burnout among paediatric nurses. J Nurs Manag. 2016;24(7):923–33. doi: 10.1111/jonm.12399 27271021

[56] Boamah SA, Read EA, Spence Laschinger HK. Factors influencing new graduate nurse burnout development, job satisfaction and patient care quality: a time-lagged study. J Adv Nurs. 2017;73(5):1182–95. doi: 10.1111/jan.13215 27878844

[57] Plevová I, Zeleníková R, Jarošová D, Janíková E. The relationship between nurse’s job satisfaction and missed nursing care. Med Pr. 2021;72(3):231–7. doi: 10.13075/mp.5893.01035 33783436

[58] Medeni V, Medeni İ, Altunay G, Dikmen AU, İlhan MN. Job satisfaction, life satisfaction, and associated factors among hospital nurses: a cross-sectional study in Türkiye. Sci Rep. 2025;15(1):5738. doi: 10.1038/s41598-025-85564-4 39962150 PMC11832888

[59] Zheng X, Song J, Shi X, Kan C, Chen C. The effect of authoritarian leadership on young nurses’ burnout: the mediating role of organizational climate and psychological capital. BMC Health Serv Res. 2025;25(1):292. doi: 10.1186/s12913-025-12403-7 39979902 PMC11844191

[60] Helaß M, Greinacher A, Genrich M, Müller A, Angerer P, Gündel H, et al. Nursing staff and supervisors perceptions on stress and resilience: a qualitative study. BMC Nurs. 2025;24(1):76. doi: 10.1186/s12912-025-02712-x 39844142 PMC11756110

