# Supplementary material for: Vicarious post-traumatic growth in Chinese oncology nurses: A cross-sectional study
Source: PLoS One. 2025 Jun 18;20(6):e0326185. doi: 10.1371/journal.pone.0326185 (PMC12176216; doi:10.1371/journal.pone.0326185)
Supplement: S1 Table — (DOCX) [file pone.0326185.s001.docx]

S1 Table Collinear diagnosis of included variables

| Variables | Standardized coefficient  Beta | T | Significance | Collinearity Statisties | |
| --- | --- | --- | --- | --- | --- |
|  |  |  |  | Toleranee | VIF |
| Age: 30-39 y | 0.003 | 0.029 | 0.977 | 0.170 | 5.867 |
| Age: ≥40 y | -0.116 | -0.816 | 0.415 | 0.107 | 9.315 |
| Job Experience: 6-15y | 0.013 | 0.116 | 0.907 | 0.165 | 6.071 |
| Job Experience: ≥15y | 0.018 | 0.123 | 0.903 | 0.105 | 9.494 |
| Job title: Intermediate | -0.067 | -0.935 | 0.351 | 0.415 | 2.409 |
| Job title: Associate chief or chief nurse | 0.066 | 1.002 | 0.317 | 0.498 | 2.009 |
| Education: Bachelor degree | 0.012 | 0.205 | 0.837 | 0.632 | 1.582 |
| Education: Master degree | -0.018 | -0.315 | 0.753 | 0.680 | 1.470 |
| Marital status | -0.045 | -0.460 | 0.646 | 0.226 | 4.420 |
| Fertility status | 0.190 | 1.866 | 0.063 | 0.208 | 4.819 |
| VT | -0.062 | -1.253 | 0.211 | 0.886 | 1.129 |
| Social support | 0.132 | 2.549 | 0.011 | 0.804 | 1.244 |
| Job satisfaction | 0.264 | 5.198 | 0.000 | 0.834 | 1.199 |

Dependent variable: Vicarious Post-Traumatic Growth.

VIF: variance inflation factors.

Collinearity exists if the tolerance is <0.1 or the VIF is >10
