# Supplementary material for: Vicarious post-traumatic growth in Chinese oncology nurses: A cross-sectional study
Source: PLoS One. 2025 Jun 18;20(6):e0326185. doi: 10.1371/journal.pone.0326185 (PMC12176216; doi:10.1371/journal.pone.0326185)

The psychometric analysis of the VTQ and C-PTGI

Psychometric analysis of the VTQ:

The internal consistency of the VTQ subscales and total scale (n=401)

| Scale Name | Number of items | Internal consistency reliability (Cronbach's α) | |
| --- | --- | --- | --- |
|  |  | α | Standardized α |
| negative affect | 6 | 0.898 | 0.898 |
| avoidance and somatization | 12 | 0.957 | 0.958 |
| negative cognition and alert reaction | 11 | 0.923 | 0.923 |
| total scale | 29 | 0.957 | 0.959 |

Assessment of Factor Model Fit in the VTQ Exploratory Factor Analysis(n=401)

| KMO | 0.960 | |
| --- | --- | --- |
| Bartlett | χ² | 9078.497 |
|  | df | 406 |
|  | *P* | 0.000 |


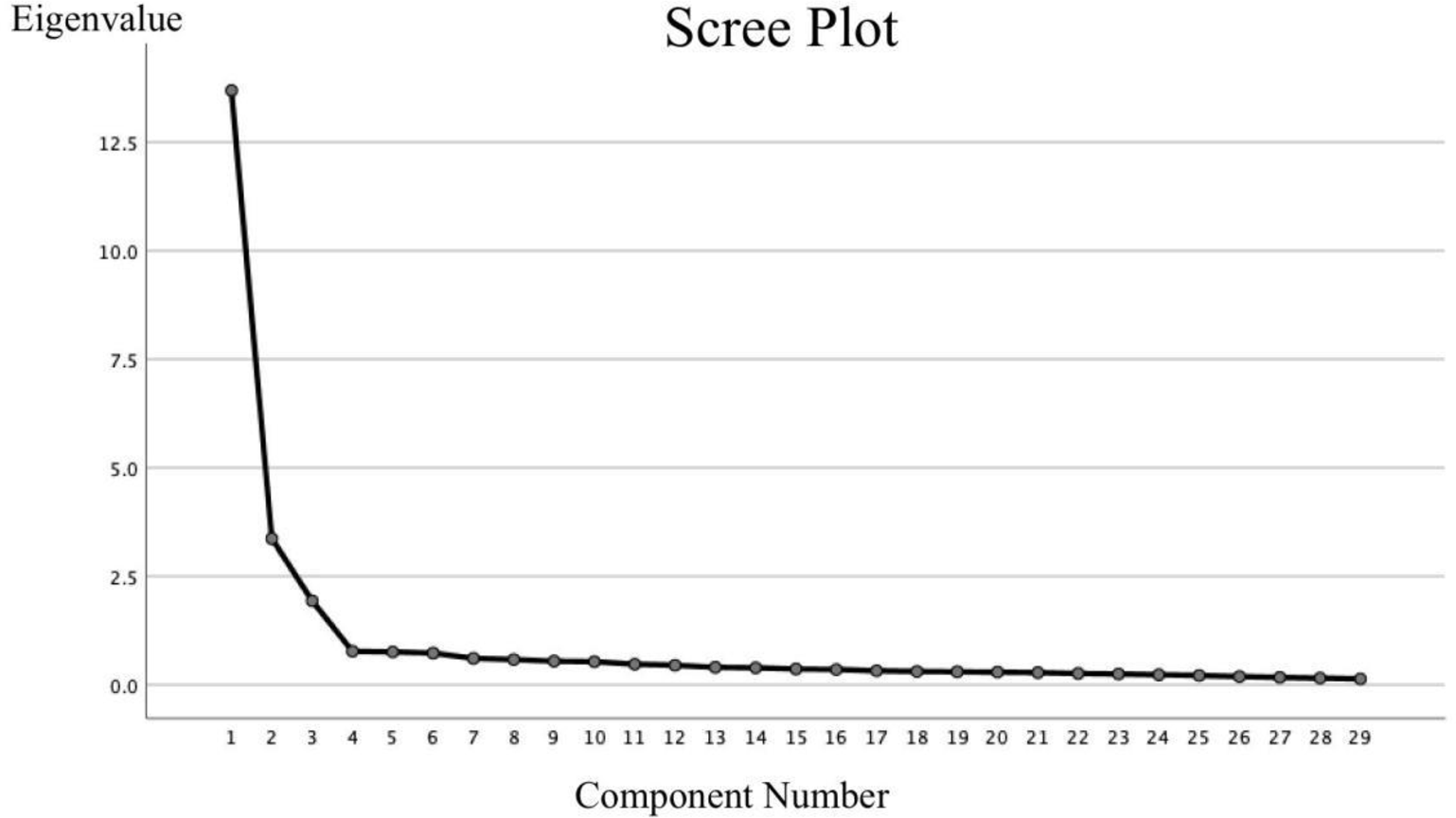


Rotated Factor Pattern Matrix for VTQ Exploratory Factor Analysis

| item | Factor 1 | Factor 2 | Factor 3 |
| --- | --- | --- | --- |
| item1 | -0.028 | 0.153 | **0.74** |
| item2 | 0.297 | 0.264 | **0.682** |
| item3 | 0.193 | 0.225 | **0.767** |
| item4 | 0.366 | 0.3 | **0.672** |
| item5 | 0.277 | 0.3 | **0.775** |
| item6 | 0.273 | 0.331 | **0.736** |
| item7 | **0.683** | 0.204 | 0.414 |
| item8 | **0.672** | 0.226 | 0.377 |
| item9 | **0.68** | 0.246 | 0.288 |
| item10 | **0.75** | 0.196 | 0.329 |
| item11 | **0.798** | 0.076 | 0.142 |
| item12 | **0.772** | 0.328 | 0.24 |
| item13 | **0.788** | 0.227 | 0.082 |
| item14 | **0.861** | 0.167 | 0.16 |
| item15 | **0.841** | 0.191 | 0.122 |
| item16 | **0.812** | 0.239 | 0.064 |
| item17 | **0.817** | 0.138 | 0.084 |
| item18 | **0.818** | 0.248 | 0.117 |
| item19 | 0.376 | **0.46** | 0.317 |
| item20 | 0.171 | **0.801** | 0.142 |
| item21 | 0.302 | **0.597** | 0.248 |
| item22 | 0.09 | **0.726** | 0.314 |
| item23 | 0.247 | **0.642** | 0.194 |
| item24 | 0.116 | **0.808** | 0.077 |
| item25 | 0.304 | **0.647** | 0.296 |
| item26 | 0.145 | **0.797** | 0.177 |
| item27 | 0.21 | **0.593** | 0.254 |
| item28 | 0.135 | **0.776** | 0.157 |
| item29 | 0.427 | **0.633** | 0.215 |

VTQ Confirmatory Factor Analysis(CFA)

| CMIN/DF | GFI | CFI | TLI | RMSEA |
| --- | --- | --- | --- | --- |
| 2.926 | 0.838 | 0.921 | 0.912 | 0.069 |


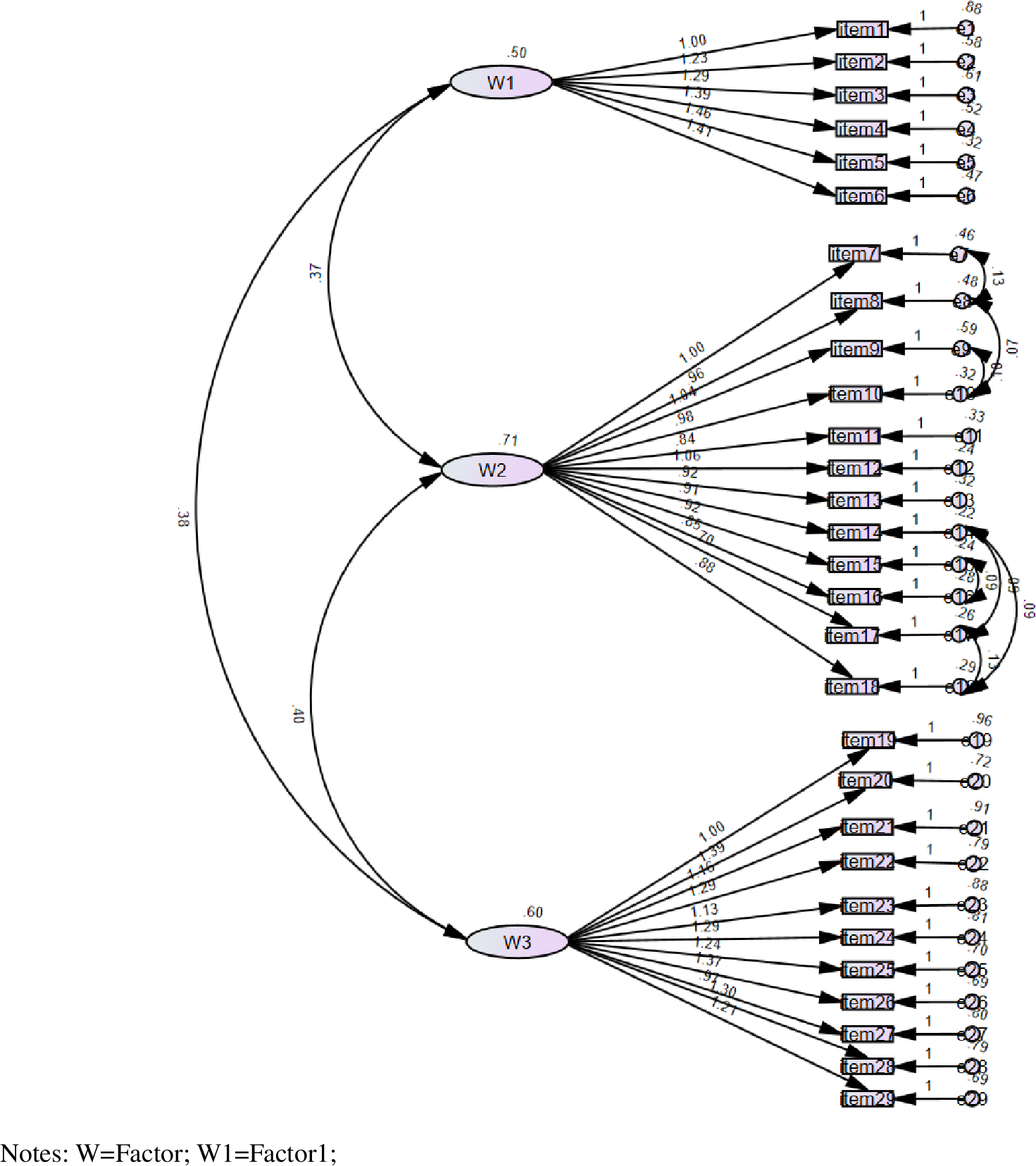


Psychometric analysis of the C-PTGI:

The internal consistency of the C-PTGI total scale (n=401)

| Scale Name | Number of items | Internal consistency reliability (Cronbach's α) | |
| --- | --- | --- | --- |
|  |  | α | Standardized α |
| total scale | 20 | 0.965 | 0.966 |

C-PTGI Confirmatory Factor Analysis(CFA) (n=401)

| CMIN/DF | GFI | CFI | TLI | RMSEA |
| --- | --- | --- | --- | --- |
| 5.841 | 0.799 | 0.897 | 0.879 | 0.110 |


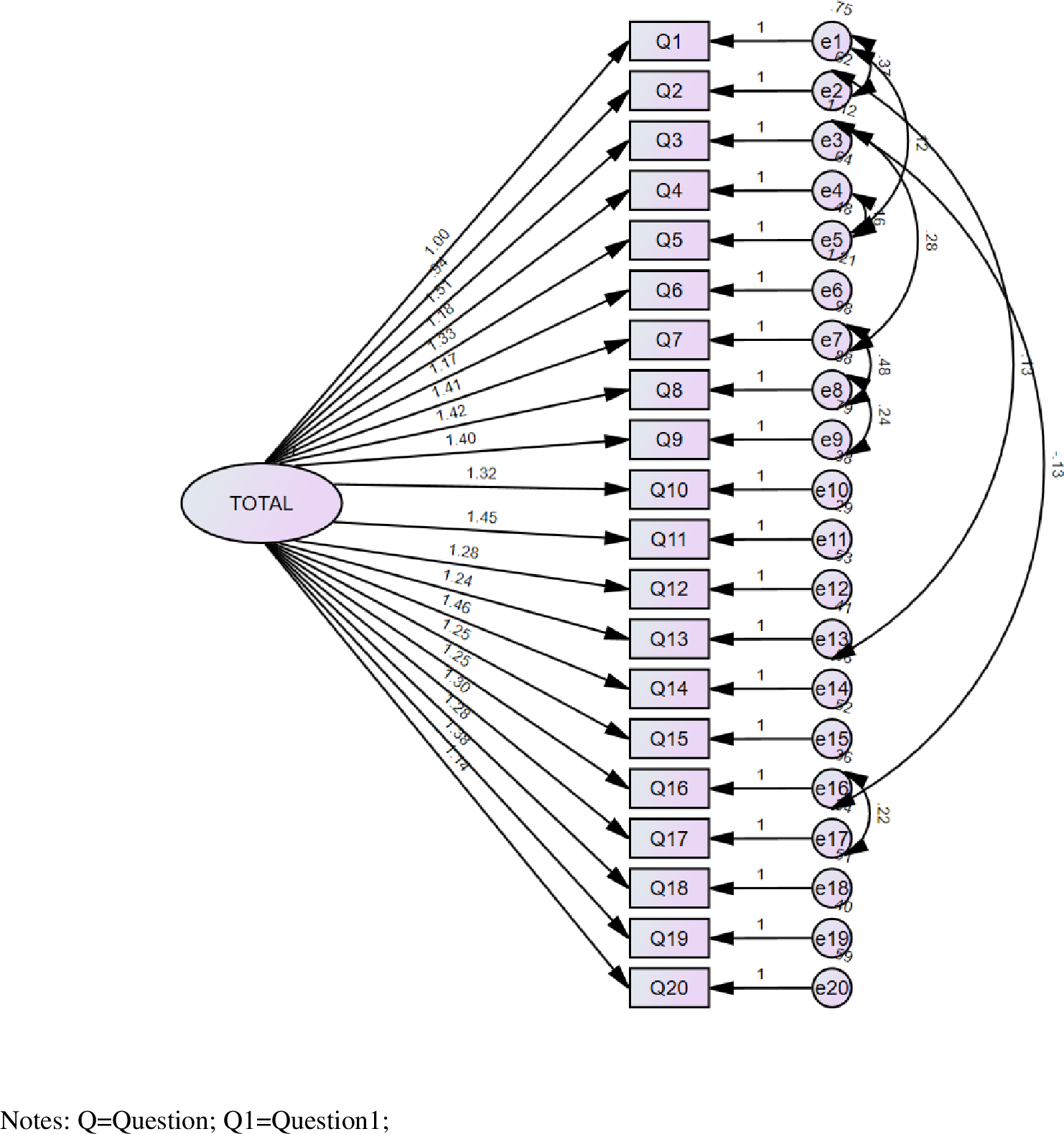

Supplement: S1 Appendix — (DOCX) [file pone.0326185.s002.docx]
